# Change in children’s physical activity and sedentary time between Year 1 and Year 4 of primary school in the B-PROACT1V cohort

**DOI:** 10.1186/s12966-017-0492-0

**Published:** 2017-04-28

**Authors:** Russell Jago, Emma Solomon-Moore, Corrie Macdonald-Wallis, Simon J. Sebire, Janice L. Thompson, Deborah A. Lawlor

**Affiliations:** 1 Centre for Exercise, Nutrition & Health Sciences, School for Policy Studies, University of Bristol, 8 Priory Road, Bristol, BS8 1TZ UK; 2 School of Sport, Exercise and Rehabilitation Sciences, University of Birmingham, Birmingham, B15 2TT UK; 3 MRC Integrative Epidemiology Unit at the University of Bristol, Oakfield House, Oakfield Grove, Bristol, BS8 2BN UK; 4 School of Social and Community Medicine, University of Bristol, Canynge Hall, Whiteladies Road, Bristol, BS8 2PS UK

**Keywords:** Physical activity, Children, Cohort, Longitudinal

## Abstract

**Background:**

The aim of this study was to examine how children’s and parents’ physical activity changes from Year 1 (5–6) to Year 4 (8–9 years of age).

**Methods:**

Data are from the Bristol (UK) B-PROACT1V cohort. Fifty-seven primary schools were recruited when the children were in Year 1, with 1299 children and their parents providing data. Forty-seven schools were re-recruited in Year 4, with 1223 children and parents providing data (685 of whom participated in Year 1). Children and at least one parent wore an accelerometer for 5 days including a weekend and mean minutes of sedentary time, moderate-to-vigorous intensity physical activity (MVPA) and accelerometer counts per minute (CPM) were derived. Multiple imputation was used to impute missing data for all 1837 families who took part, including those who participated at just one time. Paired t-tests examined if there was statistical evidence of change in accelerometer measures.

**Results:**

Multiple imputation and observed data were comparable and results using complete observed data were mostly the same as those using imputed data. Imputed data showed that mean boys’ CPM decreased from 747 to 673 (difference in mean 74 [95% CI 45 to 103]) and girls’ from 686 to 587 (99 [79 to 119]). Boys’ time spent in MVPA reduced from 72 to 69 (3 [0 to 6]) and girls’ from 62 to 56 (7 [4 to 9]) minutes per day. There were increases in sedentary time for both boys (354 to 428 min, 74 [61 to 88]) and girls (365 to 448, 83 [71 to 96]). There was no evidence of change in parent CPM or MVPA. Mothers’ sedentary time increased by 26 min per day [16 to 35].

**Conclusions:**

There were similar increases in sedentary time in girls and boys between age 5–6 and 8–9, and decreases in MVPA that were more marked in girls. The similarity of multiple-imputed and complete observed data suggest that these findings may not be markedly affected by selection bias. Result support early interventions to prevent the age-related decline in children’s physical activity.

**Electronic supplementary material:**

The online version of this article (doi:10.1186/s12966-017-0492-0) contains supplementary material, which is available to authorized users.

## Background

Physical activity during childhood has been shown to moderately track into adulthood [1], and is associated with lower levels of a number of risk factors for cardiovascular disease and type 2 diabetes including insulin, glucose, blood pressure and body composition [2]. Physical activity is also associated with improved emotional well-being and self-esteem among young people [3]. Data from the UK Millennium cohort study showed that only 51% of 7-year-old children met the recommendation of an hour of moderate-to-vigorous intensity physical activity (MVPA) per day [4]. Thus, there is a need to understand children’s physical activity patterns, how they change during maturation and key ages in which to target behaviour change interventions.

A number of studies have examined how children’s physical activity and sedentary time change as they age [5–7]. The most comprehensive analysis is the pooled data from 20 studies and 27,637 participants included in the International Children’s Accelerometry Database (ICAD), which showed an average decrease of 4.2% in total physical activity with each additional year of age [8]. However, despite the strength of the database, the majority of the studies include children from age eight or older with data for younger children based on cross-sectional studies [8]. As such, the ICAD data are limited on the information they can provide in terms of within-person change in physical activity during the early years of schooling. It is also important to highlight that several studies have reported that age-related changes in physical activity are not consistent for weekdays and weekend days. For example, the Speedy study showed a marked decrease in weekend MVPA after the transition from primary to secondary school that was not apparent for weekdays [6]. Thus, there is a need to study the within-child changes in physical activity and differences between weekdays and weekend days at the start of primary school.

Two studies have examined within-person change in younger children’s physical activity. A New Zealand study with 242 children has shown that children at age 5 engaged in approximately 50% of the physical activity that they took part in at age 3 [9]. Physical activity patterns at age 7 were comparable to those at age 5, and boys were more active than girls at all ages [9]. Analysis of a cohort of 300 UK children reported that there was little change in physical activity between 5 and 8 years of age before declining progressively between 9 and 15 years of age [10]. Both of these studies are, however, comparatively small and as such there is a need for larger studies that can provide more representative data.

While there is evidence children’s physical activity patterns decline as they age [8, 11] it is not clear if parental physical activity patterns change as children get older. We have previously reported weak associations between parent and child physical activity [12–14] suggesting that parents may not need to be active with their children to promote physical activity and reduce sedentary time [15, 16]. However, becoming a parent has been associated with a decline in physical activity [17, 18] with parents of young children reporting new responsibilities and time commitments as barriers to physical activity [19–21] and a shift in priorities from themselves to their child [22]. It may be reasonable to assume, therefore, that as children age and become more independent, parents are able to re-address this balance and find more time to participate in their own physical activity. To the best of our knowledge, there is, however a lack of information on whether parental physical activity patterns change as children move through primary school. Understanding change in parents’ physical activity as children age would be helpful for identifying time-points when parent and child activity patterns may be more or less amenable to behaviour change interventions.

The aims of this paper were to examine the change in children’s physical activity between 5–6 and 8–9 years of age in a UK-based cohort and if there were any differences by gender or day of the week. Secondly, as changes in the start of primary school may affect parents’ opportunities for physical activity, we also examined if there were within-person changes in parents’ physical activity over the same period.

## Methods

### Study design

**Fig. 1:**
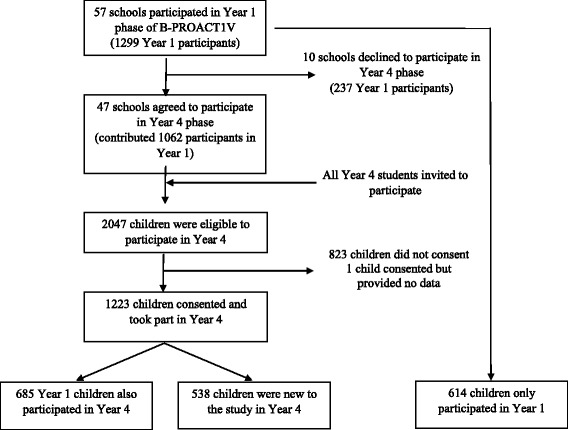
Flow diagram of recruitment to the Year 4 phase of the B-PROACT1V study (STROBE)

The current analyses used data from a longitudinal study (B-PROACT1V) conducted at the University of Bristol (UK). The aim of the study was to examine the physical activity and sedentary behaviours of children and their parents during primary school. Extensive detail on the first phase of data collection has been previously published [13, 23]. Briefly, study recruitment began in January 2012, with data collection conducted between February 2012 and July 2013 when the children were in their second year of schooling (known as Year 1 in the UK – children were aged 5 to 6 years). Two hundred fifty primary schools within Bristol, Bath and North Somerset were invited to take part in the study, from which 57 schools consented to participate and data collection was conducted. All children in Year 1 (or Y1 and Y2 in schools with combined classes) were eligible, with 1299 children and at least one of their parents consenting to participate (see Fig. 1).

The second phase of data collection was conducted when the children were in Year 4 (aged 8 to 9 years) between March 2015 and July 2016. All 57 schools that participated in the first phase were invited to participate in the second phase; 10 schools declined for various reasons (e.g., Ofsted (government) inspections, staff changes, scheduling issues), with the remaining 47 schools agreeing to participate. Where possible, schools were recruited in the same order as the Year 1 data collection to closely replicate the data collection timeline and average difference between the dates of the Year 1 and 4 data collections was 6.9 days. The median difference in age between the time of data collection for each phase for children who took part in Year 1 and Year 4 was 3.00 years and 95% of the age differences ranged between 2.79 and 3.65 years. All children in Year 4 (or Y4 and Y5 in schools with combined classes) were eligible (*n* = 2047) regardless of whether they had participated in the first phase of data collection. In total, 1223 (59.7%) children and at least one of their parents consented and took part in the Year 4 data collection. One family consented but was not available for data collection.

### Data collection at Year 4

Researchers arranged to visit each school to conduct a briefing presentation with the Year 4 children to explain the study. After the presentation, children were given an information pack to take home to their parents/carers. Child participation in the study was dependent on at least one parent or carer (maximum of two parents/carers) also agreeing to participate in the study. Ethical approval for the study was granted from the School for Policy Studies Research Ethics Committee at the University of Bristol and written parental consent was provided for both parent and child participation [24].

Child height was measured to the nearest 0.1 cm using a SECA Leicester stadiometer (HAB International, Northampton). Weight was recorded to the nearest 0.1 kg using a SECA 899 digital scale (HAB International, Northampton). The children were then given a waist-worn ActiGraph wGT3X accelerometer, shown how and when to wear it, and given a pack to take home to their parents.

Parent packs contained either one or two accelerometers, depending on the number of parents/carers participating. Parents received instructions on how and when to wear the accelerometer. If indicated on the consent form, the packs also contained paper versions of the parent questionnaires. Alternatively, parents were sent a link to a secure online version of the questionnaire. The parent questionnaires assessed demographic variables and a number of psychosocial constructs that are not reported here.

Children and parents were instructed to wear the accelerometers for five full days (3 week days and 2 weekend days). During data collection a mobile phone SMS reminder system was in operation to inform parents about the when the accelerometers and questionnaires were being sent home and when to return the devices. At the end of the 5 days, parents were instructed to return the accelerometers and completed paper questionnaires to a marked returns box at the child’s school. If accelerometers or questionnaires were not returned directly, parents were sent reminder texts, calls and/or emails. If after 2 weeks, devices or questionnaires were still outstanding a letter and prepaid envelope were sent directly to the child’s home address. As a thank you for participating children were given a water bottle and a Frisbee upon completion of data collection.

### Accelerometer data processing

Accelerometer data were processed using Kinesoft (v3.3.75; Kinesoft, Saskatchewan, Canada) and each day was considered valid if there was at least 500 min of data after excluding intervals ≥60 min of zero counts allowing up to two minutes of interruptions. For the complete case analysis, at least one valid weekday and at least one valid weekend day of data were required at both the Year 1 and Year 4 assessments. To maximise the sample size, if a participant had at least one valid day of data at either time point, this partial data was included in the imputation models (see below) to provide an indication of physical activity for the participant. Average counts per minute (CPM), average number of sedentary minutes per day and average number of MVPA minutes per day overall and separately by weekdays and weekend days for the children and their parents were derived. Minutes spent in MVPA were derived using population-specific cut points for children and adults [25, 26].

### Child characteristics

Child gender and the number of siblings were reported by the parent. An age-adjusted BMI z-score was derived using the 1990 UK child growth reference, and categorised as under/normal weight (<85th percentile), overweight (≥85th percentile) or obese (≥ 95th percentile) [27]. Indices of Multiple Deprivation (IMD) scores, based upon the English Indices of Deprivation (http://data.gov.uk/dataset/index-of-multiple-deprivation), were assigned to each child-parent dyad based on their reported home postcode where higher IMD scores indicate a greater level of deprivation. Each school was asked to provide information on whether the child had moved school or remained in the same school between Year 1 and Year 4 (three schools refused to provide this information). The parent questionnaire at Year 4 also asked whether the child had moved school between Year 1 and Year 4.

### Parent characteristics

Parent gender, age, height, weight, ethnicity and employment status were reported in the two parental questionnaires. Body mass index was calculated from self-reported height and weight (BMI = kg/m^2^).

### Statistical analysis

Child and parent characteristics measured during the Year 1 phase of B-PROACT1V were examined as potential predictors of the child’s participation in the Year 4 phase using univariable logistic regression models. Odds ratios for participation at Year 4 versus not participating are presented for each characteristic.

To enable us to include information from all study participants in our analysis, and thus potentially increase statistical power and precision of estimates of change in physical activity, we used multiple imputation of missing data. This method also allows demographic factors that are predictive of missingness (but are not necessarily required in the analysis model) to be accounted for in the imputation procedure and can therefore reduce selection bias compared with analysis including only individuals with complete data [28]. We imputed data for the 1837 children who participated in either Year 1 or Year 4 using chained equations; this included imputing complete Year 4 data if the child participated in Year 1 but not Year 4 and vice versa. Twenty imputed datasets were created using 20 cycles of regression switching and results were then averaged over these datasets using Rubin’s rules [29]. Separate imputation models were used for boys and girls to allow for possible differences in missing data patterns that would influence results and to allow exploration of different patterns of change in physical activity between Year 1 and Year 4 by child gender. All child and parent accelerometer measures and child and parent characteristics that were potential predictors of missingness (child BMI, IMD and number of siblings and female/male parent response, age, BMI, ethnicity and employment status) at either year, were included in multiple imputation models. We also included a categorical variable indicating which school the child attended in order to account for the clustering of children within schools. For the three children who attended a different school at Year 4 from that in Year 1, we used their Year 1 school as this seemed most likely to influence their physical activity change between Year 1 and Year 4. The distributions of all included variables have been compared in the observed data and in the multiple imputation datasets.

The children’s physical activity levels (mean and SD) in Year 1 and Year 4 and the change in these between Year 1 and Year 4 were summarised for the imputed datasets and are presented separately for boys and girls. Confidence intervals for the change in physical activity were derived using robust standard errors to account for clustering of children within schools. Paired *t*-tests (based on these robust standard errors) were used to assess whether there was statistical evidence of a change in physical activity between Year 1 and Year 4. The female and male parents’ physical activity levels in Year 1 and Year 4 were also summarised and compared in the same way. Additionally, the mean and SD of each of the physical activity measures at Year 1 and Year 4 have been summarised for children and parents in the complete case analyses and the change in these measures was assessed, as in the imputed data, using paired t tests with robust standard errors.

There was a high degree of missing data for the male parents since male parents were less likely to participate in any aspect of the study than female parents. To check whether this was affecting our findings, we also imputed to the 864 children and parent triads where a male parent responded at either Year 1 or Year 4 and repeated the analysis of male parents’ physical activity levels in this subgroup as a sensitivity analysis. All analyses were performed in Stata version 14.0 (StataCorp, 2015).

## Results

**Fig. 2:**
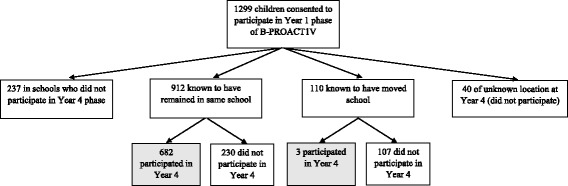
Follow up of children who participated in the Year 1 phase of the B-PROACT1V study

Of the 1837 children who participated in the study at either year, 614 (33.4%) participated in Year 1 only, 538 (29.3%) participated in Year 4 only and 685 (37.3%) participated in both Year 1 and Year 4. Figure 2 shows the Year 4 school locations (same school, different study school, different non-study school) of the 1299 children who took part in Year 1, 52.7% of whom were successfully re-recruited to the study in Year 4. Those re-recruited were mostly children who remained in the same school between the two study years, although three children moved within study schools and were still able to participate at both time points.

Table 1 shows the characteristics of all of the children and parents who participated at Year 1 and at Year 4, as well as the subset who participated in both time points. The distributions of these characteristics were broadly similar at each phase and within the group who participated at both phases, with the exception of the percentage of children having moved school between Year 1 and Year 4, which was much lower in the group that participated in both years, as expected. In both Year 1 and Year 4, children were more likely to have a female parent take part in the study than a male parent. The male parents were slightly older on average than the female parents and a higher proportion were in full time employment. The distributions of child and parent accelerometer measures and demographic characteristics in the multiple imputation datasets and in the observed data are compared in Additional file 1: Table S1. There was little difference in these between the observed and imputed data, except that the proportion of non-White British and not working parents was higher in the imputed data than in the observed data.

**Table 1:** Characteristics of the children and parents who participated in B-PROACT1V in Year 1, Year 4 and both years

Child Characteristic		Participated in Year 1 (*N* = 1,299)		Participated in Year 4 (*N* = 1,223)		Participated in both Year 1 and Year 4 (*N* = 685)			
						Year 1		Year 4	
		N with data		N with data		N with data		N with data	
Gender N (%)	Boy	1299	666 (51.3)	1223	556 (45.5)	685	323 (47.2)	*As for Year 1*	*As for Year 1*
	Girl		633 (48.7)		667 (54.5)		362 (52.8)		
BMI age-adjusted z-score Mean (SD)		1024	0.24 (0.93)	962	0.32 (1.07)	570	0.18 (0.91)	554	0.28 (1.04)
IMD score Median (IQR)		1171	10.8 (6.2, 19.4)	1204	11.1 (6.6, 21.7)	627	10.5 (6.1, 18.9)	673	10.4 (5.7, 20.8)
Moved schools between Year 1 and Year 4 (school information) N (%)	No	1022	912 (89.2)			685	682 (99.6)		
	Yes		110 (10.8)				3 (0.4)		
Moved schools between Year 1 and Year 4 (parent questionnaire) N (%)	No			1000	874 (87.4)			573	551 (96.2)
	Yes				126 (12.6)				22 (3.8)
Number of siblings N (%)	0	791	36 (4.6)	996	173 (17.4)	432	19 (4.4)	570	91 (16.0)
	1		471 (59.5)		498 (50.0)		275 (63.7)		307 (53.9)
	2		215 (27.2)		232 (23.3)		110 (25.5)		129 (22.6)
	3 or more		69 (8.7)		93 (9.3)		28 (6.5)		43 (7.5)
Female parent responded N (%)	No	1299	414 (31.9)	1223	420 (34.3)	685	217 (31.7)	685	212 (30.9)
	Yes		885 (68.1)		803 (65.7)		468 (68.3)		473 (69.1)
Male parent responded N (%)	No	1299	775 (59.7)	1223	733 (59.9)	685	400 (58.4)	685	401 (58.5)
	Yes		524 (40.3)		490 (40.1)		285 (41.6)		284 (41.5)
Female parent age (years) Mean (SD)		839	37.3 (5.5)	740	40.8 (6.0)	457	37.6 (5.6)	439	40.7 (5.7)
Male parent age (years) Mean (SD)		498	39.8 (5.8)	382	43.2 (6.1)	274	40.1 (6.0)	218	43.6 (5.8)
Female parent BMI (kg/m^2^) Mean (SD)		844	25.0 (4.5)	761	25.8 (5.2)	450	24.8 (4.3)	451	25.4 (4.8)
Male parent BMI (kg/m^2^) Mean (SD)		511	26.3 (3.9)	475	26.4 (3.9)	280	26.1 (3.8)	278	26.4 (3.8)
Female parent ethnicity N (%)	White British	885	788 (89.0)	802	707 (88.2)	592	527 (89.0)	*As for Year 1*	*As for Year 1*
	White Other		44 (5.0)		47 (5.9)		31 (5.2)		
	Black/Black British		14 (1.6)		8 (1.0)		7 (1.2)		
	Asian/Asian British		20 (2.3)		23 (2.9)		18 (3.0)		
	Mixed		10 (1.1)		9 (1.1)		6 (1.0)		
	Other		9 (1.0)		8 (1.0)		3 (0.5)		
Male parent ethnicity N (%)	White British	521	465 (89.3)	490	436 (89.0)	419	380 (90.7)	*As for Year 1*	*As for Year 1*
	White Other		22 (4.2)		21 (4.3)		15 (3.6)		
	Black/Black British		3 (0.6)		5 (1.0)		3 (0.7)		
	Asian/Asian British		16 (3.1)		16 (3.3)		12 (2.9)		
	Mixed		6 (1.2)		8 (1.6)		5 (1.2)		
	Other		9 (1.7)		4 (0.8)		4 (1.0)		
Female parent employment status N (%)	Working full time	882	133 (15.1)	803	198 (24.7)	466	81 (17.4)	473	109 (23.0)
	Working part-time		488 (55.3)		438 (54.5)		256 (54.9)		274 (57.9)
	Full time parent/care-giver		193 (21.9)		121 (15.1)		95 (20.4)		65 (13.7)
	In full time education		13 (1.5)		8 (1.0)		3 (0.6)		5 (1.1)
	Not currently employed		55 (6.2)		38 (4.7)		31 (6.7)		20 (4.2)
Male parent employment status N (%)	Working full time	517	462 (89.4)	490	454 (92.7)	281	256 (91.1)	284	264 (93.0)
	Working part-time		28 (5.4)		23 (4.7)		11 (3.9)		10 (3.5)
	Full time parent/care-giver		10 (1.9)		3 (0.6)		5 (1.8)		2 (0.7)
	In full time education		3 (0.6)		1 (0.2)		2 (0.7)		1 (0.4)
	Not currently employed		14 (2.7)		9 (1.8)		7 (2.5)		7 (2.5)

The associations of each of the characteristics of children and their parents measured at Year 1 with their participation in the study in Year 4 are shown in Table 2. Girls who participated at Year 1 were more likely to participate in Year 4 than boys, and children who were obese at Year 1 were less likely to participate at Year 4 than those who were underweight/normal weight.

**Table 2:** Associations of child and parent characteristics at Year 1 with the odds of child participation in the study in Year 4

Child Characteristic at Year 1 (Total *N* = 1,299)		Child participated in Year 4
		Odds ratio (95% CI)
Child gender	Boy	Reference
	Girl	1.42 (1.07, 1.88)
BMI category	Under/Normal weight	Reference
	Overweight	0.78 (0.55, 1.10)
	Obese	0.63 (0.39, 1.03)
IMD score		0.99 (0.97, 1.01)
Number of siblings	0	Reference
	1	1.26 (0.62, 2.53)
	2	0.94 (0.47, 1.87)
	3 or more	0.61 (0.26, 1.44)
Female parent responded	No	Reference
	Yes	1.02 (0.82, 1.27)
Male parent responded	No	Reference
	Yes	1.12 (0.87, 1.44)
Female parent age (years)		1.03 (0.99, 1.06)
Male parent age (years)		1.02 (0.99, 1.05)
Female parent BMI (kg/m^2^)		0.98 (0.95, 1.01)
Male parent BMI (kg/m^2^)		0.98 (0.93, 1.03)
Female parent ethnicity	White British	Reference
	Other	0.82 (0.54, 1.25)
Male parent ethnicity	White British	Reference
	Other	0.81 (0.49, 1.35)
Female parent employment status	Not working	Reference
	Working full/part time or in full time education	1.12 (0.82, 1.52)
Male parent employment status	Not working	Reference
	Working full/part time or in full time education	1.20 (0.53, 2.70)

The average CPM, sedentary minutes per day and MVPA minutes per day at Year 1 and Year 4, and change in these, for all boys and girls using multiple imputation are shown in Table 3. In both boys and girls there was strong evidence that mean CPM over all days decreased between Year 1 and Year 4 while sedentary minutes per day increased overall. There was also strong evidence in girls, and slightly weaker evidence in boys, that MVPA per day decreased overall between Year 1 and Year 4. When analysed separately by weekday and weekend day, there was strong evidence for reductions in CPM and increases in sedentary time on weekdays and weekend days in both boys and girls, as well as for reductions in MVPA on weekdays and weekend days in girls. However, there was only weak evidence for a reduction in MVPA on weekdays in boys and no difference in MVPA on weekend days. Similar patterns of association were seen in the complete case analyse and these results were consistent with the multiple imputation analysis (Additional file 2: Table S2).

**Table 3:** Change in child physical activity accelerometer measures between Year 1 and Year 4 by gender for all who participated at Year 1 or Year 4 using multiple imputation of missing values (*N* = 1837)^a^

Physical Activity Measure	Boys (*N* = 899)				Girls (*N* = 938)			
	Year 1	Year 4	Change Year 1 to Year 4		Year 1	Year 4	Change Year 1 to Year 4	
	Mean (SD)	Mean (SD)	Mean (95% CI)	*P* for difference^*^	Mean (SD)	Mean (SD)	Mean (95% CI)	*P* for difference^*^
Counts per minute overall	747.1 (187.3)	673.1 (211.8)	−74.0 (−103.3, −44.7)	<0.001	685.6 (155.2)	586.8 (186.5)	−98.8 (−119.0, −78.6)	<0.001
Counts per minute on a weekday	735.1 (184.8)	660.7 (197.0)	−74.4 (−106.7, −42.1)	<0.001	670.6 (152.1)	570.7 (158.4)	−99.9 (−122.3, −77.6)	<0.001
Counts per minute on a weekend day	769.7 (296.8)	705.9 (351.8)	−63.8 (−120.9, −6.8)	0.03	715.3 (255.9)	614.1 (339.6)	−101.2 (−137.9, −64.5)	<0.001
Average sedentary minutes per day overall	354.1 (58.6)	428.2 (106.6)	74.2 (60.5, 87.9)	<0.001	364.8 (59.7)	448.2 (107.3)	83.4 (70.8, 96.0)	<0.001
Average sedentary minutes per weekday	362.9 (63.4)	442.4 (111.6)	79.5 (63.3, 95.7)	<0.001	377.5 (66.2)	462.0 (112.1)	84.5 (70.5, 98.5)	<0.001
Average sedentary minutes per weekend day	339.0 (75.3)	405.3 (122.2)	66.3 (47.2, 85.5)	<0.001	340.6 (74.4)	423.5 (118.3)	82.9 (66.7, 99.1)	<0.001
Average MVPA minutes per day overall	72.4 (20.7)	69.3 (22.7)	−3.0 (−6.4, 0.3)	0.07	62.3 (16.2)	55.6 (17.3)	−6.7 (−9.0, −4.4)	<0.001
Average MVPA minutes per weekday	73.2 (22.2)	69.6 (22.8)	−3.6 (−7.2, 0.0)	0.05	62.6 (17.8)	56.6 (18.8)	−6.0 (−8.6, −3.4)	<0.001
Average MVPA minutes per weekend day	70.9 (28.6)	69.0 (34.7)	−1.8 (−7.2, 3.6)	0.49	61.6 (22.4)	53.6 (25.4)	−8.0 (−11.4, −4.5)	<0.001

^a^78 children had no valid accelerometer data at either time point; 596 had valid data at Year 1 but not Year 4 (456 complete), 557 had valid data at Year 4 but not Year 1 (439 complete); 606 had valid data at both time points (67 complete for Year 4 only, 70 complete for Year 1 only, 446 complete for both years (included in Additional file 2: Table S2)

^*^ *P*-value obtained from a paired *t*-test that the difference in the means of the Year 1 and Year 4 values is 0, using robust standard errors to account for clustering by school

The change in CPM, sedentary minutes per day and MVPA per day for the parents of all children who took part in either Year 1 or Year 4 using multiple imputation is shown in Table 4. There was little evidence that CPM or MVPA changed over the 3 years for female or male parents, or that sedentary time changed in male parents, whether using overall measures or analysing these separately by weekday and weekend day. However, there was evidence of an increase in sedentary minutes per day overall, as well as in weekdays and weekend days separately, in female parents. In analysis including only the complete cases findings were consistent with the multiple imputation analysis as confidence intervals were wide (Additional file 3: Table S3). However, in this small subset of the data there was an increase in MVPA in female and male parents between Year 1 and Year 4 that was not seen in the imputed data.

**Table 4:** Change in parent physical activity accelerometer measures between Year 1 and Year 4 for all who participated at Year 1 or Year 4 using multiple imputation of missing values (*N* = 1837)^a^

Physical Activity Measure	Female parent (*N* = 1837)				Male parent (*N* = 1837)			
	Year 1	Year 4	Change Year 1 to Year 4		Year 1	Year 4	Change Year 1 to Year 4	
	Mean (SD)	Mean (SD)	Mean (95% CI)	*P* for difference^*^	Mean (SD)	Mean (SD)	Mean (95% CI)	*P* for difference^*^
Counts per minute overall	405.9 (141.4)	399.5 (139.5)	−6.4 (−17.2, 4.4)	0.23	409.1 (144.0)	419.8 (164.8)	10.7 (−14.2, 35.5)	0.37
Counts per minute on a weekday	420.0 (141.9)	415.0 (141.7)	−5.0 (−17.5, 7.4)	0.42	415.3 (167.6)	421.3 (178.6)	6.1 (−30.2, 42.3)	0.72
Counts per minute on a weekend day	383.1 (217.7)	372.9 (210.5)	−10.2 (−30.8, 10.3)	0.31	397.2 (256.2)	423.2 (257.2)	26.0 (−40.4, 92.4)	0.39
Average sedentary minutes per day overall	509.2 (77.1)	535.0 (106.1)	25.8 (16.2, 35.3)	<0.001	546.0 (92.1)	555.5 (113.3)	9.5 (−11.8, 30.9)	0.34
Average sedentary minutes per weekday	523.0 (91.4)	547.9 (118.9)	24.9 (13.5, 36.3)	<0.001	565.8 (117.2)	571.0 (133.2)	5.2 (−22.0, 32.5)	0.68
Average sedentary minutes per weekend day	485.8 (101.5)	510.7 (128.1)	24.9 (5.8, 43.9)	0.01	518.3 (127.1)	528.4 (160.2)	10.1 (−25.3, 45.6)	0.54
Average MVPA minutes per day overall	48.2 (19.7)	48.5 (20.1)	0.3 (−1.1, 1.8)	0.67	51.0 (21.7)	54.1 (24.1)	3.0 (−0.2, 6.2)	0.06
Average MVPA minutes per weekday	52.7 (24.3)	52.6 (24.7)	−0.1 (−2.1, 1.9)	0.94	54.5 (28.7)	56.8 (32.3)	2.3 (−2.6, 7.1)	0.33
Average MVPA minutes per weekend day	41.1 (26.5)	41.6 (25.8)	0.5 (−2.5, 3.5)	0.73	45.6 (36.7)	49.4 (37.6)	3.8 (−8.8, 16.4)	0.49

1016 children had no valid male parent accelerometer data; 361 had valid data for Year 1 but not Year 4 (321 complete); 323 had valid data for Year 4 but not Year 1 (281 complete); 137 had valid data at both time points (6 complete for Year 4 only, 9 complete for Year 1 only, 122 complete in both years (included in Additional file 3: Table S3))

^a^558 children had no valid female parent accelerometer data; 524 had valid data for Year 1 but not Year 4 (456 complete); 436 had valid data for Year 4 but not Year 1 (379 complete); 319 had valid data at both time points (21 complete for Year 4 only, 21 complete for Year 1 only, 268 complete in both years (included in Additional file 3: Table S3))

^*^ *P*-value obtained from a paired *t*-test that the difference in the means of the Year 1 and Year 4 values is 0, using robust standard errors to account for clustering by school

In the sensitivity analysis in which we repeated the analysis of physical activity levels in male parents, imputing missing data only for families in which the male parent responded at either Year 1 or Year 4, findings were consistent with the main analysis.

## Discussion

The data presented in this paper have shown that there are important changes in children’s physical activity between Year 1 and Year 4 of primary school. Accelerometer CPM decreased by 10% for boys and 8.6% for girls, time spent in MVPA by 3 min (4%) for boys and 7 min (11%) for girls, while sedentary time increased by 20% for boys and 23% for girls. Overall, the findings indicate that while there were important changes for both genders, the magnitude and absolute change in MVPA was more marked for girls than boys. These patterns were broadly consistent when comparing weekdays and weekend days except for boys’ weekend MVPA, which did not differ between the two assessment points. With the exception of mothers’ sedentary time, which increased by 25 min (5%) per day over the 3-year period, there was no evidence that parents’ physical activity patterns changed between the two assessment periods. The data presented here therefore show marked changes, in an undesirable direction (with regards to their health), in children’s physical activity and sedentary time as the children move through primary school that are not reflected among their parents.

The within-child declines in physical activity and associated increases in sedentary time between Year 1 and Year 4 are broadly consistent with the ICAD data which showed an average decrease of 4.2% in total physical activity with each additional year of age [8]. The findings are also consistent with the results of a meta-analysis of self-reported adolescent physical activity which identified a decrease of 7% in physical activity per year [30]. The analysis in this paper extends these findings to show that the age-related decline in MVPA is evident during the early years of school. Importantly, our data also shows a very substantial increase in sedentary time between Year 1 and 4, indicating that the children are not just less active, they are also spending more time being sedentary. Collectively these findings highlight a need to identify the factors that are contributing to both the decrease in MVPA and also the increase in sedentary time with age, as both may be useful targets for strategies to attenuate the decline in physical activity.

Girls who participated in Year 1 were more likely to take part in the Year 4 assessment than boys, and children who were a healthy weight in Year 1 were more likely to participate in Year 4 than children who were obese. This finding is comparable to previous analyses of the ALSPAC cohort which reported that children attending the clinics at ages 12, 14 and 16 were more likely to be girls and from a higher social class [31]. Similarly, the Speedy study reported that participants included in the analysis of change in physical activity during the move from primary to secondary school were more likely to be girls and from more affluent households, but they found no differences by anthropometric assessments [6]. However, analysis of the ROOTS study reported that participants who provided data at age 15 and 17 who provided data at both time points had a lower fat mass index (as measured by bio-electrical impedance) at age 15 than those who only provided data at age 15 [32]. These findings are important as they suggest that bias could be introduced by restricting the sample to a “complete case” dataset.

A key issue in accelerometer studies is how “missing” accelerometer data are handled and the implications that those decisions have on sample bias. Prospective studies that include objective measures of physical activity often include a high level of missing data because either participants were not present at both assessments (attrition), or they provided only partial data at one of the assessments. Partial data is a particular issue for accelerometer-based physical activity studies in which a participant is required to provide a number of “valid days” at each time point, with 3 days often used as a criteria [33, 34]. For example in the PEACH project, accelerometer data were provided by 1307 children at the end of primary school (10–11 years of age), and of these, 953 (73%) children provided data a year later when in the first year of secondary school (11–12 years of age) [35]. However, due to missing data, the analysis sample was further reduced to 518 participants, with 290 (30.4%) of those with data at both time points excluded due to incomplete accelerometer data [35]. The reduction in the sample size for these analyses has the potential to introduce selection bias to coefficient estimates as it may be the case that there are some participant characteristics such as baseline level of physical activity, body mass, or socio-economic position that are related to data provision [28, 36, 37]. By not taking account of these important differences between the target population and the analysis sample, the interpretation of study findings can be biased and the data presented in this paper suggest that by limiting the analysis sample to complete case we would have introduced this kind of error.

In the analyses reported in this paper, we used multiple imputation methods to create a complete dataset for 1837 children and their parents, and the complete case and imputed dataset were broadly comparable with the exception of parent ethnicity and employment status. We recognise that there are limitations to the multiple imputation approach. In particular, the method assumes that data are missing at random (MAR) and thus any differences between individuals whose data are observed and those whose data are missing can be explained by observed variables and these must be included in the imputation model [28]. However, if data are missing not at random (MNAR) and there are reasons for missingness that depend on unobserved data, once observed data are taken into account, then the multiple imputation approach will provide biased estimates and this bias increases with the proportion of missing data [38]. In our analysis data may be MNAR if, for example, children who experienced a large decline in physical activity between Year 1 and Year 4 were less likely to participate at Year 4. We are unable to assess from the observed data whether the MAR assumption holds, however by including a wide range of child and parent characteristics that were predictive of missingness in our multiple imputation model we aimed to reduce the possibility of data being MNAR and minimise bias in our estimates of change in physical activity. In our study, it is plausible that the multiple imputation approach reduced selection bias compared with the complete case analysis since it allowed for the participants’ BMI, school, and socio-economic position to be accounted for despite these not being required in the analysis model and therefore not incorporated in complete case analysis. It also facilitated the creation of a markedly larger sample size to increase precision in estimates of physical activity change. Previous studies have suggested that at least 3 days of valid accelerometer data are required to provide an indication of habitual physical activity patterns [33, 34], but we have included accelerometer data for anyone with at least one valid day in order to maximise the use of the data. A key issue for the field is, however, how multiple imputation methods and partial accelerometer data can be optimally combined to provide the largest possible sample size while also providing the most accurate representation of habitual physical activity.

The four UK Chief Medical Officers recommend that all children and adolescents should engage in an hour per day of MVPA [39], and this recommendation has recently been highlighted as a key component of the English childhood obesity strategy [40]. Our imputed analyses showed that Year 4 boys engaged in an average of 69 min of MVPA per day, but for girls the mean was 55 min of MVPA per day. Further examination of these data showed that 62.3% of boys and 35.5% of girls met the hour per day recommendation in Year 4. This was compared with 72.5% of boys and 53.7% of girls in Year 1. As there is clear evidence that children’s physical activity declines with age [8, 11] with girls less active than boys at all ages, these findings highlight a need to develop effective means of increasing children’s physical activity and attenuating the age-related decline in activity during the primary school years.

### Strengths and limitations

The key strength of this project is the provision of detailed physical activity and related information from children as they move through primary school and their parents, which has facilitated a detailed examination of how physical activity behaviours have changed within individuals. Moreover, we have demonstrated that we can maximise the information available to create a dataset of 1837 children and their families using multiple imputation approaches. The study, is however, limited by ten of the original schools not participating in the follow-up assessment. The relatively homogenous sample, which is primarily of White British origin from a single UK area, also limits the ability to extrapolate to other ethnic groups in more diverse areas of the UK. Where possible, the data collection timelines were closely matched between the two time points, however for some participants data was collected in different months of the year, meaning that weather effects and day length may have had an affect on physical activity levels [41]. However, when we repeated analysis restricting only to participants from schools where the data collection at Year 4 was within 1 month of the data collection at Year 1 (67% of participants) the findings were essentially unchanged from those presented here.

## Conclusions

Between Year 1 and Year 4 of primary school accelerometer counts per minute decreased by 10% for boys and 8.6% for girls. Minutes of MVPA decreased by 3 min (4%) for boys and 7 min (11%) for girls, and sedentary time increased by 20% for boys and 23% for girls. Findings suggest that early intervention to prevent the age-related decline in children’s physical activity is needed and work to identify key effective interventions is an urgent public health need.

## Additional files

Additional file 1: Table S1. Comparison of observed data and multiple imputation datasets for all variables included in the multiple imputation models. (DOC 109 kb)

Additional file 2: Table S2. Change in child physical activity accelerometer measures between Year 1 and Year 4 by gender for those who had complete accelerometer data at both years (*N* = 446). (DOC 38 kb)

Additional file 3: Table S3. Change in parent physical activity accelerometer measures between Year 1 and Year 4 for those who had complete accelerometer data at both years (*N* = 268 female parents; *N* = 122 male parents). (DOC 39 kb)

## Abbreviations

BMI: Body mass index
CPM: Accelerometer counts per minute
IMD: Index of Multiple Deprivation
MVPA: Moderate to Vigorous intensity Physical Activity
SD: Standard Deviation
UK: United Kingdom
