# Supplementary material for: Change in children’s physical activity and sedentary time between Year 1 and Year 4 of primary school in the B-PROACT1V cohort
Source: Int J Behav Nutr Phys Act. 2017 Apr 28;14:33. doi: 10.1186/s12966-017-0492-0 (PMC5408437; doi:10.1186/s12966-017-0492-0)
Supplement: Supplementary file 1 — Comparison of observed data and multiple imputation datasets for all variables included in the multiple imputation models. (DOC 109 kb) [file 12966_2017_492_MOESM1_ESM.doc]

**Additional file 1: Table S1.** Comparison of observed data and multiple imputation datasets for all variables included in the multiple imputation models

|  | | | | **Observed Data** | | **Multiple Imputation Datasets used for Overall PA measures (N=1837)** |
| --- | --- | --- | --- | --- | --- | --- |
|  |  |  |  | **N** | **Mean (SD) or %** | **Mean (SD) or %** |
| **Child Characteristics** | | | | | | |
| Counts per minute overall in Year 1 | | | | 1202 | 718.0 (185.1) | 715.7 (174.4) |
| Counts per minute overall in Year 4 | | | | 1163 | 618.6 (203.7) | 629.1 (203.9) |
| Counts per minute on a weekday in Year 1 | | | | 1194 | 704.1 (186.4) | 702.2 (171.9) |
| Counts per minute on a weekday in Year 4 | | | | 1155 | 600.6 (190.1) | 614.7 (183.9) |
| Counts per minute on a weekend in Year 1 | | | | 980 | 745.4 (273.7) | 741.9 (278.0) |
| Counts per minute on a weekend in Year 4 | | | | 960 | 656.8 (330.7) | 659.0 (348.8) |
| Average sedentary minutes overall in Year 1 | | | | 1202 | 359.3 (61.0) | 359.6 (59.4) |
| Average sedentary minutes overall in Year 4 | | | | 1163 | 440.2 (112.8) | 438.4 (107.4) |
| Average sedentary minutes per weekday in Year 1 | | | | 1194 | 370.2 (65.8) | 370.3 (65.3) |
| Average sedentary minutes per weekday in Year 4 | | | | 1155 | 454.8 (114.7) | 452.4 (112.3) |
| Average sedentary minutes per weekend day in Year 1 | | | | 980 | 339.1 (76.0) | 339.8 (74.9) |
| Average sedentary minutes per weekend day in Year 4 | | | | 960 | 416.3 (133.6) | 414.6 (120.6) |
| Average MVPA minutes overall in Year 1 | | | | 1202 | 67.6 (20.7) | 67.2 (19.2) |
| Average MVPA minutes overall in Year 4 | | | | 1163 | 62.1 (22.6) | 62.3 (21.2) |
| Average MVPA minutes per weekday in Year 1 | | | | 1194 | 68.1 (21.7) | 67.8 (20.7) |
| Average MVPA minutes per weekday in Year 4 | | | | 1155 | 62.4 (22.7) | 63.0 (21.8) |
| Average MVPA minutes per weekend day in Year 1 | | | | 980 | 66.3 (27.6) | 66.1 (26.0) |
| Average MVPA minutes per weekend day in Year 4 | | | | 960 | 61.3 (32.0) | 61.1 (31.3) |
| Child gender | | | Boy | 1837 | 48.9 | 48.9 |
|  |  |  | Girl |  | 51.1 | 51.1 |
| BMI age-adjusted z-score at Year 1 | | | | 1024 | 0.24 (0.93) | 0.25 (1.03) |
| BMI age-adjusted z-score at Year 4 | | | | 962 | 0.32 (1.07) | 0.41 (1.19) |
| Log of IMD score at Year 1 | | | | 1171 | 2.38 (0.82) | 2.40 (0.83) |
| Log of IMD score at Year 4 | | | | 1204 | 2.41 (0.88) | 2.46 (0.89) |
| Number of siblings at Year 1 | | | 0 | 791 | 4.6 | 11.4 |
|  |  |  | 1 |  | 59.5 | 49.0 |
|  |  |  | 2 |  | 27.2 | 25.1 |
|  |  |  | 3 or more |  | 8.7 | 14.6 |
| Number of siblings at Year 4 | | | 0 | 996 | 17.4 | 19.3 |
|  |  |  | 1 |  | 50.0 | 45.7 |
|  |  |  | 2 |  | 23.3 | 22.7 |
|  |  |  | 3 or more |  | 9.3 | 12.3 |
| **Female parent characteristics** | | | | | | |
| Counts per minute overall in Year 1 | | | | 843 | 406.6 (148.0) | 405.9 (141.4) |
| Counts per minute overall in Year 4 | | | | 755 | 398.5 (147.8) | 399.5 (139.5) |
| Counts per minute on a weekday in Year 1 | | | | 835 | 420.6 (161.9) | 420.0 (141.9) |
| Counts per minute on a weekday in Year 4 | | | | 748 | 409.7 (163.8) | 415.0 (141.7) |
| Counts per minute on a weekend in Year 1 | | | | 753 | 380.0 (176.9) | 383.1 (217.7) |
| Counts per minute on a weekend in Year 4 | | | | 675 | 372.9 (162.9) | 372.9 (210.5) |
| Average sedentary minutes overall in Year 1 | | | | 843 | 508.3 (83.2) | 509.2 (77.1) |
| Average sedentary minutes overall in Year 4 | | | | 755 | 532.2 (115.5) | 535.0 (106.1) |
| Average sedentary minutes per weekday in Year 1 | | | | 835 | 524.1 (92.3) | 523.0 (91.4) |
| Average sedentary minutes per weekday in Year 4 | | | | 748 | 549.7 (122.6) | 547.9 (118.9) |
| Average sedentary minutes per weekend day in Year 1 | | | | 753 | 486.4 (97.3) | 485.8 (101.5) |
| Average sedentary minutes per weekend day in Year 4 | | | | 675 | 507.5 (130.9) | 510.7 (128.1) |
| Average MVPA minutes overall in Year 1 | | | | 843 | 48.1 (22.9) | 48.2 (19.7) |
| Average MVPA minutes overall in Year 4 | | | | 755 | 48.7 (23.7) | 48.5 (20.1) |
| Average MVPA minutes per weekday in Year 1 | | | | 835 | 52.9 (26.4) | 52.7 (24.3) |
| Average MVPA minutes per weekday in Year 4 | | | | 748 | 52.7 (27.2) | 52.6 (24.7) |
| Average MVPA minutes per weekend day in Year 1 | | | | 753 | 40.2 (26.0) | 41.1 (26.5) |
| Average MVPA minutes per weekend day in Year 4 | | | | 675 | 41.1 (24.4) | 41.6 (25.8) |
| Female parent responded at Year 1 | | | No | 1299 | 31.9 | 35.0 |
|  |  |  | Yes |  | 68.1 | 65.0 |
| Female parent responded at Year 4 | | | No | 1223 | 34.3 | 38.3 |
|  |  |  | Yes |  | 65.7 | 61.7 |
| Female parent age at Year 1 (years) | | | | 839 | 37.3 (5.5) | 37.3 (6.3) |
| Female parent age at Year 4 (years) | | | | 740 | 40.8 (6.0) | 40.6 (6.6) |
| Female parent BMI at Year 1 (kg/m^2^) | | | | 844 | 25.0 (4.5) | 25.2 (5.3) |
| Female parent BMI at Year 4 (kg/m^2^) | | | | 761 | 25.8 (5.2) | 26.0 (6.2) |
| Female parent ethnicity | White British | | | 1339 | 88.3 | 82.3 |
|  | Other | | |  | 11.7 | 17.7 |
| Female parent employment status at Year 1 | | Not working | | 882 | 28.1 | 32.5 |
|  |  | Working/education | |  | 71.9 | 67.5 |
| Female parent employment status at Year 4 | | Not working | | 803 | 19.8 | 28.6 |
|  |  | Working/education | |  | 80.2 | 71.4 |
| **Male parent characteristics** | | | | | | |
| Counts per minute overall in Year 1 | | | | 498 | 410.6 (160.8) | 409.1 (144.0) |
| Counts per minute overall in Year 4 | | | | 460 | 418.2 (167.0) | 419.8 (164.8) |
| Counts per minute on a weekday in Year 1 | | | | 492 | 417.0 (185.1) | 415.3 (167.6) |
| Counts per minute on a weekday in Year 4 | | | | 458 | 417.4 (189.4) | 421.3 (178.6) |
| Counts per minute on a weekend in Year 1 | | | | 458 | 392.5 (185.5) | 397.2 (256.2) |
| Counts per minute on a weekend in Year 4 | | | | 411 | 414.4 (198.8) | 423.2 (257.2) |
| Average sedentary minutes overall in Year 1 | | | | 498 | 546.3 (88.8) | 546.0 (92.1) |
| Average sedentary minutes overall in Year 4 | | | | 460 | 556.5 (114.7) | 555.5 (113.3) |
| Average sedentary minutes per weekday in Year 1 | | | | 492 | 567.0 (104.3) | 565.8 (117.2) |
| Average sedentary minutes per weekday in Year 4 | | | | 458 | 575.6 (124.4) | 571.0 (133.2) |
| Average sedentary minutes per weekend day in Year 1 | | | | 458 | 521.0 (92.0) | 518.3 (127.1) |
| Average sedentary minutes per weekend day in Year 4 | | | | 411 | 527.4 (130.5) | 528.4 (160.2) |
| Average MVPA minutes overall in Year 1 | | | | 498 | 51.7 (25.4) | 51.0 (21.7) |
| Average MVPA minutes overall in Year 4 | | | | 460 | 54.8 (27.0) | 54.1 (24.1) |
| Average MVPA minutes per weekday in Year 1 | | | | 492 | 55.4 (29.9) | 54.5 (28.7) |
| Average MVPA minutes per weekday in Year 4 | | | | 458 | 56.9 (32.1) | 56.8 (32.3) |
| Average MVPA minutes per weekend day in Year 1 | | | | 458 | 45.1 (27.7) | 45.6 (36.7) |
| Average MVPA minutes per weekend day in Year 4 | | | | 411 | 51.0 (31.2) | 49.4 (37.6) |
| Male parent responded at Year 1 | | | No | 1299 | 59.7 | 59.3 |
|  |  |  | Yes |  | 40.3 | 40.7 |
| Male parent responded at Year 4 | | | No | 1223 | 59.9 | 60.5 |
|  |  |  | Yes |  | 40.1 | 39.5 |
| Male parent age at Year 1 (years) | | | | 498 | 39.8 (5.8) | 39.5 (9.7) |
| Male parent age at Year 4 (years) | | | | 382 | 43.2 (6.1) | 43.7 (12.4) |
| Male parent BMI at Year 1 (kg/m^2^) | | | | 511 | 26.3 (3.9) | 26.9 (6.7) |
| Male parent BMI at Year 1 (kg/m^2^) | | | | 475 | 26.4 (3.9) | 27.1 (7.1) |
| Male parent ethnicity | | | White British | 861 | 89.2 | 74.5 |
|  |  |  | Other |  | 10.8 | 25.5 |
| Male parent employment status at Year 1 | | | Not working | 517 | 4.6 | 21.5 |
|  |  |  | Working/education |  | 95.4 | 78.5 |
| Male parent employment status at Year 4 | | | Not working | 490 | 2.4 | 25.7 |
|  | | | Working/education |  | 97.6 | 74.3 |
