# Supplementary material for: Change in children’s physical activity and sedentary time between Year 1 and Year 4 of primary school in the B-PROACT1V cohort
Source: Int J Behav Nutr Phys Act. 2017 Apr 28;14:33. doi: 10.1186/s12966-017-0492-0 (PMC5408437; doi:10.1186/s12966-017-0492-0)
Supplement: Supplementary file 2 — Change in child physical activity accelerometer measures between Year 1 and Year 4 by gender for those who had complete accelerometer data at both years (N = 446). (DOC 38 kb) [file 12966_2017_492_MOESM2_ESM.doc]

**Additional file 2: Table S2. Change in child physical activity accelerometer measures between Year 1 and Year 4 by gender for those who had complete accelerometer data at both years (N=446)**

| **Physical Activity Measure** | **Boys (N=204)** | | | | **Girls (N=242)** | | | |
| --- | --- | --- | --- | --- | --- | --- | --- | --- |
|  | **Year 1** | **Year 4** | **Change Year 1 to Year 4** | | **Year 1** | **Year 4** | **Change Year 1 to Year 4** | |
|  | **Mean (SD)** | **Mean (SD)** | **Mean**  **(95% CI)** | **P for difference^*^** | **Mean (SD)** | **Mean (SD)** | **Mean**  **(95% CI)** | **P for difference^*^** |
| **Counts per minute overall** | **718.7**  **(178.8)** | **658.8**  **(219.8)** | **-59.9**  **(-97.6, -22.2)** | **0.003** | **690.6**  **(157.5)** | **590.0**  **(185.8)** | **-100.5**  **(-129.2 , -71.9)** | **<0.001** |
| Counts per minute on a weekday | 710.6  (184.4) | 642.3  (202.2) | -68.3  (-106.5, -30.2) | 0.001 | 670.6  (156.4) | 562.5 (160.2) | -108.1  (-136.0, -80.2) | <0.001 |
| Counts per minute on a weekend day | 739.9  (288.0) | 694.2  (344.6) | -45.7  (-107.8, 16.4) | 0.15 | 727.5  (250.6) | 637.9  (318.8) | -89.5  (-134.9, -44.2) | <0.001 |
|  |  |  |  |  |  |  |  |  |
| **Average sedentary minutes per day overall** | **363.1**  **(52.4)** | **436.3**  **(107.8)** | **73.2**  **(55.0, 91.4)** | **<0.001** | **364.7**  **(62.9)** | **447.8**  **(98.9)** | **83.2**  **(62.7, 103.6)** | **<0.001** |
| Average sedentary minutes per weekday | 375.0  (56.1) | 452.2  (108.6) | 77.2  (57.4, 97.1) | <0.001 | 381.2  (65.5) | 464.8  (100.3) | 83.5  (62.0, 105.1) | <0.001 |
| Average sedentary minutes per weekend day | 341.7  (73.3) | 410.6  (127.9) | 69.0 (46.4, 91.6) | <0.001 | 336.0 (77.5) | 418.4  (113.7) | 82.4 (59.9, 104.9) | <0.001 |
|  |  |  |  |  |  |  |  |  |
| **Average MVPA minutes per day overall** | **70.9**  **(21.1)** | **69.3**  **(23.7)** | **-1.6**  **(-5.9, 2.8)** | **0.47** | **63.0**  **(16.7)** | **56.3**  **(17.4)** | **-6.6**  **(-9.4, -3.8)** | **<0.001** |
| Average MVPA minutes per weekday | 72.7  (22.7) | 70.2  (22.0) | -2.5  (-7.7, 2.7) | 0.34 | 63.5  (18.3) | 56.5  (18.4) | -6.9  (-10.1, -3.8) | <0.001 |
| Average MVPA minutes per weekend day | 68.2  (29.7) | 68.1  (36.7) | -0.1  (-6.6, 6.4) | 0.98 | 62.2  (22.6) | 55.8  (25.7) | -6.4  (-10.2, -2.6) | 0.002 |

**^*^**P-value obtained from a paired t-test that the difference in the means of the Year 1 and Year 4 values is 0, using robust standard errors to account for clustering by school
