# Supplementary material for: Change in children’s physical activity and sedentary time between Year 1 and Year 4 of primary school in the B-PROACT1V cohort
Source: Int J Behav Nutr Phys Act. 2017 Apr 28;14:33. doi: 10.1186/s12966-017-0492-0 (PMC5408437; doi:10.1186/s12966-017-0492-0)
Supplement: Supplementary file 3 — Change in parent physical activity accelerometer measures between Year 1 and Year 4 for those who had complete accelerometer data at both years (N = 268 female parents; N = 122 male parents). (DOC 39 kb) [file 12966_2017_492_MOESM3_ESM.doc]

**Additional file 3: Table S3.** Change in parent physical activity accelerometer measures between Year 1 and Year 4 for those who had complete accelerometer data at both years (N=268 female parents; N=122 male parents)

| **Physical Activity Measure** | **Female parent (N=268)** | | | | **Male parent (N=122)** | | | |
| --- | --- | --- | --- | --- | --- | --- | --- | --- |
|  | **Year 1** | **Year 4** | **Change Year 1 to Year 4** | | **Year 1** | **Year 4** | **Change Year 1 to Year 4** | |
|  | **Mean (SD)** | **Mean (SD)** | **Mean**  **(95% CI)** | **P for difference^*^** | **Mean (SD)** | **Mean (SD)** | **Mean**  **(95% CI)** | **P for difference^*^** |
| **Counts per minute overall** | **398.6**  **(130.4)** | **409.9**  **(141.1)** | **11.3**  **(-4.7, 27.3)** | **0.16** | **386.6**  **(132.0)** | **415.2**  **(141.2)** | **28.6**  **(-3.0, 60.1)** | **0.08** |
| Counts per minute on a weekday | 415.3  (147.6) | 421.1  (159.1) | 5.8  (-13.8, 25.5) | 0.55 | 379.9  (156.4) | 403.6  (158.7) | 23.6  (-8.8, 56.0) | 0.15 |
| Counts per minute on a weekend day | 373.7  (160.4) | 391.2  (173.0) | 17.5  (-4.5, 39.5) | 0.12 | 392.3  (181.9) | 432.0  (196.4) | 39.6  (-11.0, 90.3) | 0.12 |
|  |  |  |  |  |  |  |  |  |
| **Average sedentary minutes per day overall** | **524.7**  **(76.7)** | **540.2**  **(107.3)** | **15.5**  **(0.5, 30.5)** | **0.04** | **567.9**  **(72.2)** | **577.1**  **(110.7)** | **9.3**  **(-11.7, 30.2)** | **0.38** |
| Average sedentary minutes per weekday | 542.1  (88.7) | 559.5  (114.5) | 17.4  (1.1, 33.7) | 0.04 | 592.7  (92.5) | 605.7  (113.6) | 13.0  (-6.0, 32.0) | 0.18 |
| Average sedentary minutes per weekend day | 495.5  (90.7) | 509.3  (126.4) | 13.8  (-4.0, 31.6) | 0.12 | 533.8  (86.0) | 531.5  (132.6) | -2.3  (-32.7, 28.2) | 0.88 |
|  |  |  |  |  |  |  |  |  |
| **Average MVPA minutes per day overall** | **47.7**  **(21.4)** | **51.3**  **(22.4)** | **3.5**  **(1.2, 5.9)** | **0.004** | **49.3**  **(22.2)** | **55.7**  **(23.6)** | **6.4**  **(0.6, 12.1)** | **0.03** |
| Average MVPA minutes per weekday | 53.1  (24.8) | 55.5  (25.9) | 2.3  (-0.8, 5.5) | 0.15 | 50.8  (26.1) | 56.4  (28.0) | 5.6  (-0.3, 11.6) | 0.06 |
| Average MVPA minutes per weekend day | 39.6  (24.0) | 44.2  (26.1) | 4.6  (1.6, 7.6) | 0.004 | 46.6  (28.4) | 54.4  (30.7) | 7.8  (-0.4, 16.0) | 0.06 |

**^*^**P-value obtained from a paired t-test that the difference in the means of the Year 1 and Year 4 values is 0, using robust standard errors to account for clustering by school
